# Supplementary material for: Amorphous-to-rodlet structural transition governs the interfacial functions of Aspergillus oryzae hydrophobin RolA
Source: Front Microbiol. 2026 Apr 21;17:1807699. doi: 10.3389/fmicb.2026.1807699 (PMC13139150; doi:10.3389/fmicb.2026.1807699)
Supplement: Supplementary file 1 [file Data_Sheet_1.pdf]

Amorphous-to-Rodlet Structural Transition Governs the Interfacial Functions of *Aspergillus oryzae* Hydrophobin RolA

Daiki Ida[1†], Nao Takahashi[1†], Yuki Terauchi[2], Takumi Tanaka[1], Akira Yoshimi[3,4], Hirotaka Kobayashi[5], Ken Miyazawa[6], Masaya Mitsuishi[7], Hiroshi Yabu[8], Keietsu Abe[1]*

[1] Graduate School of Agricultural Science, Tohoku University, Sendai, Japan

[2] Research Center for Thermotolerant Microbial Resources (RCTMR), Yamaguchi University, Yamaguchi, Japan

[3] Graduate School of Agriculture, Kyoto University, Kyoto, Japan

[4] Graduate School of Global Environmental Studies, Kyoto University, Kyoto, Japan

[5] Department of Infectious Disease Pathology, National Institute of Infectious Diseases, Japan Institute for Health Security, Tokyo, Japan

[6] Department of Fungal Infection, National Institute of Infectious Diseases, Japan Institute for Health Security, Tokyo, Japan

[7] Graduate School of Engineering, Tohoku University, Sendai, Japan

[8] Advanced Institute for Materials Research (WPI-AIMR), Tohoku University, Sendai, Japan

***Correspondence: Keietsu Abe** keietsu.abe.b5@tohoku.ac.jp

[†]These authors contributed equally to this work and share first authorship.

Figure S1

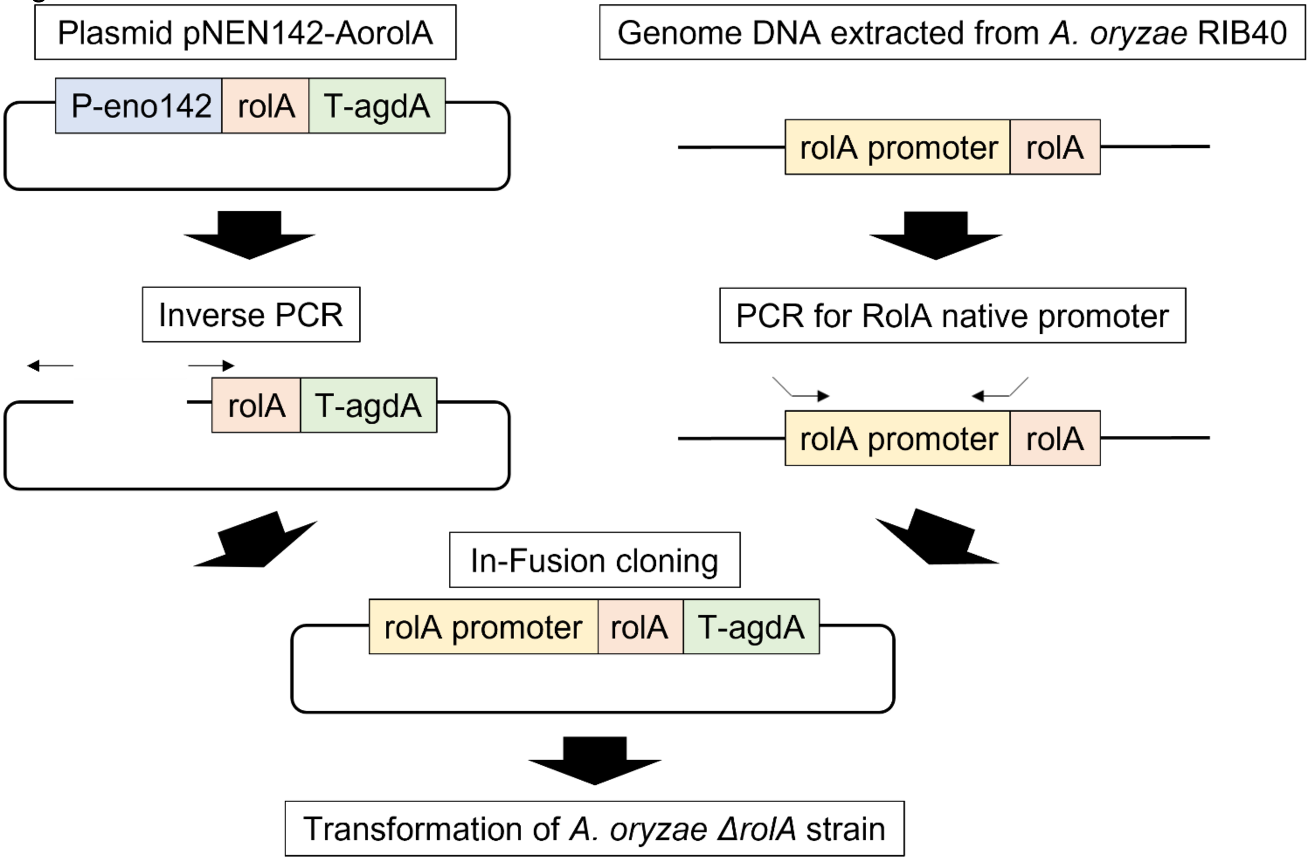

Construction of *A. oryzae* RolA-complemented strains. A plasmid expressing the RolA ORF (WT, L137S, L142S, L137S/L142S) under the control of the native promoter from the *A. oryzae* RIB40 strain was created and transformed into the *niaD* locus of the *ΔrolA* strain (parental strain: *adeA*⁻).

Figure S2

(A)

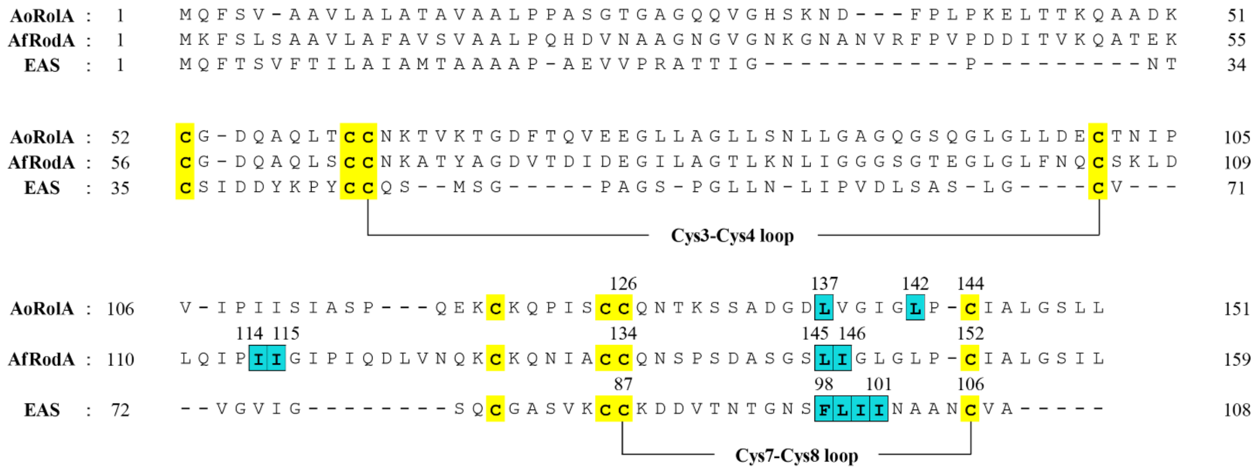

(B)

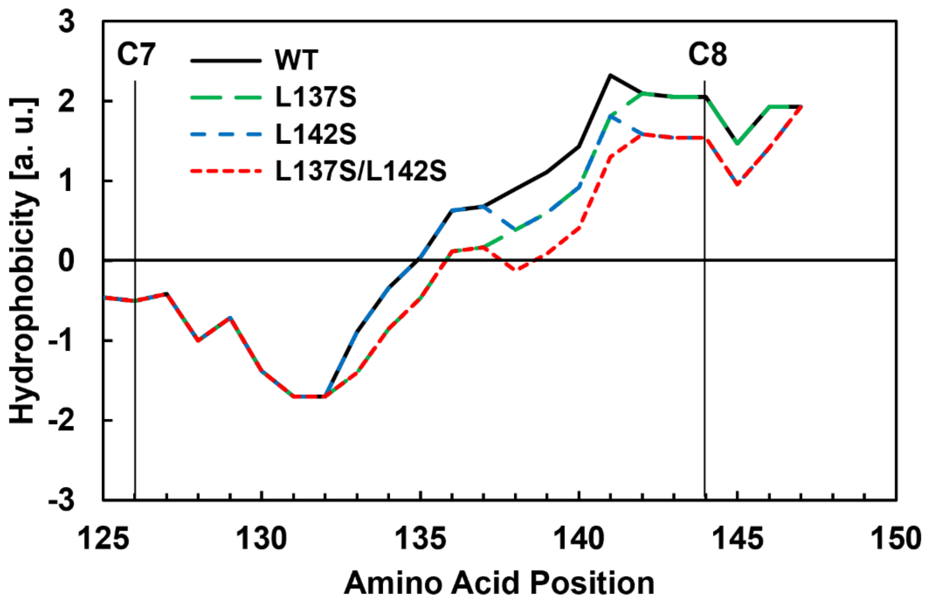

(A) Alignment of the amino acid sequences of three hydrophobins: *A. oryzae* RolA, *A. fumigatus* RodA, and *N. crassa* EAS. Cysteine residues that form conserved disulfide bonds are shown in yellow. Hydrophobic amino acid residues of RolA mutated in this study and those involved in rodlet formation in RodA and EAS are shown in cyan. Alignment was generated in CLUSTALW v. 2.1 (https://www.genome.jp/tools-bin/clustalw).

(B) Comparison of hydropathy plots of the Cys7–Cys8 loop among RolA-WT and mutants. The plot was generated by the ProtScale tool from Expasy (Expert Protein Analysis System; https://web.expasy.org/cgi-bin/protscale/protscale.pl).

Figure S3

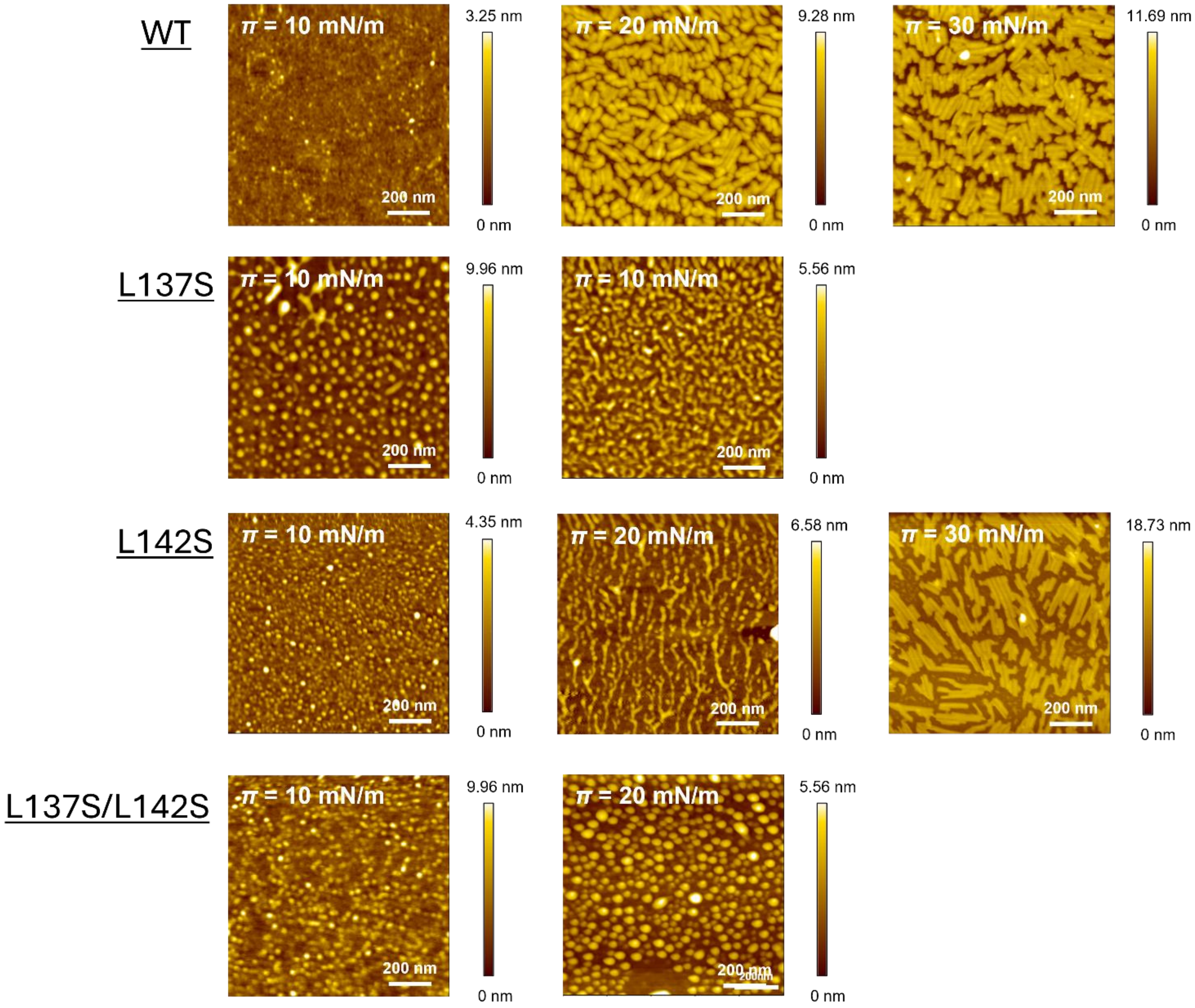

AFM topography images and height profiles of Langmuir films of wild-type RolA (WT) and its mutants. Films were transferred on hydrophobic silicon substrate at the indicated values of π. Image size, 1 μm × 1 μm.

Figure S4

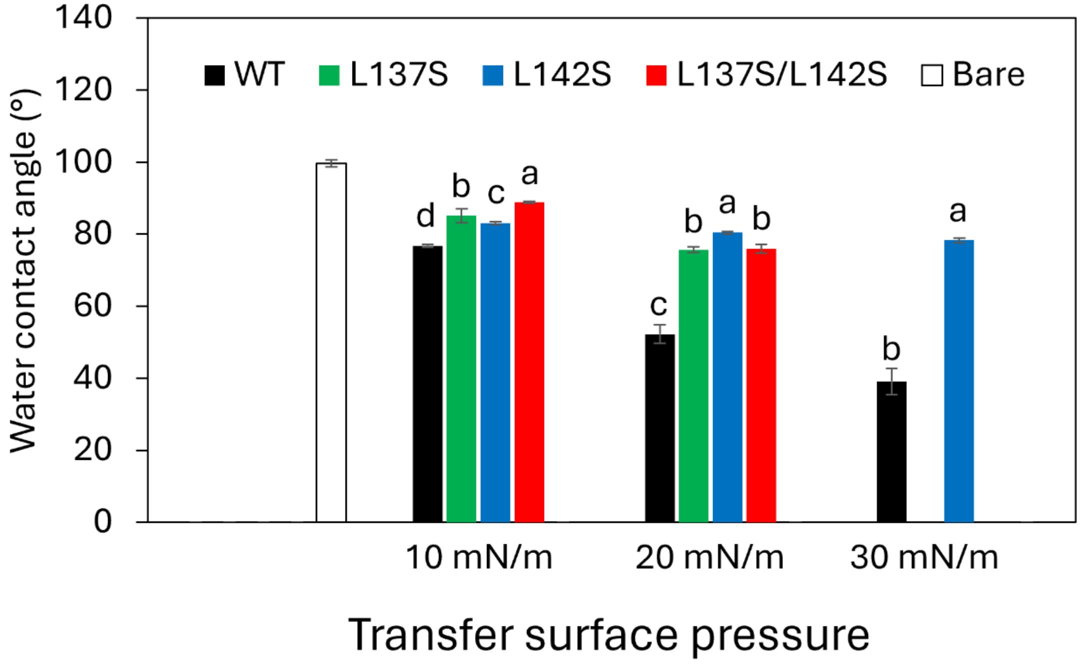

Water contact angles of Langmuir films of RolA WT and its mutants transferred onto hydrophobic substrate. Different letters denote significant differences in Tukey's test (*P* < 0.05). Error bars, standard deviations.

Figure S5

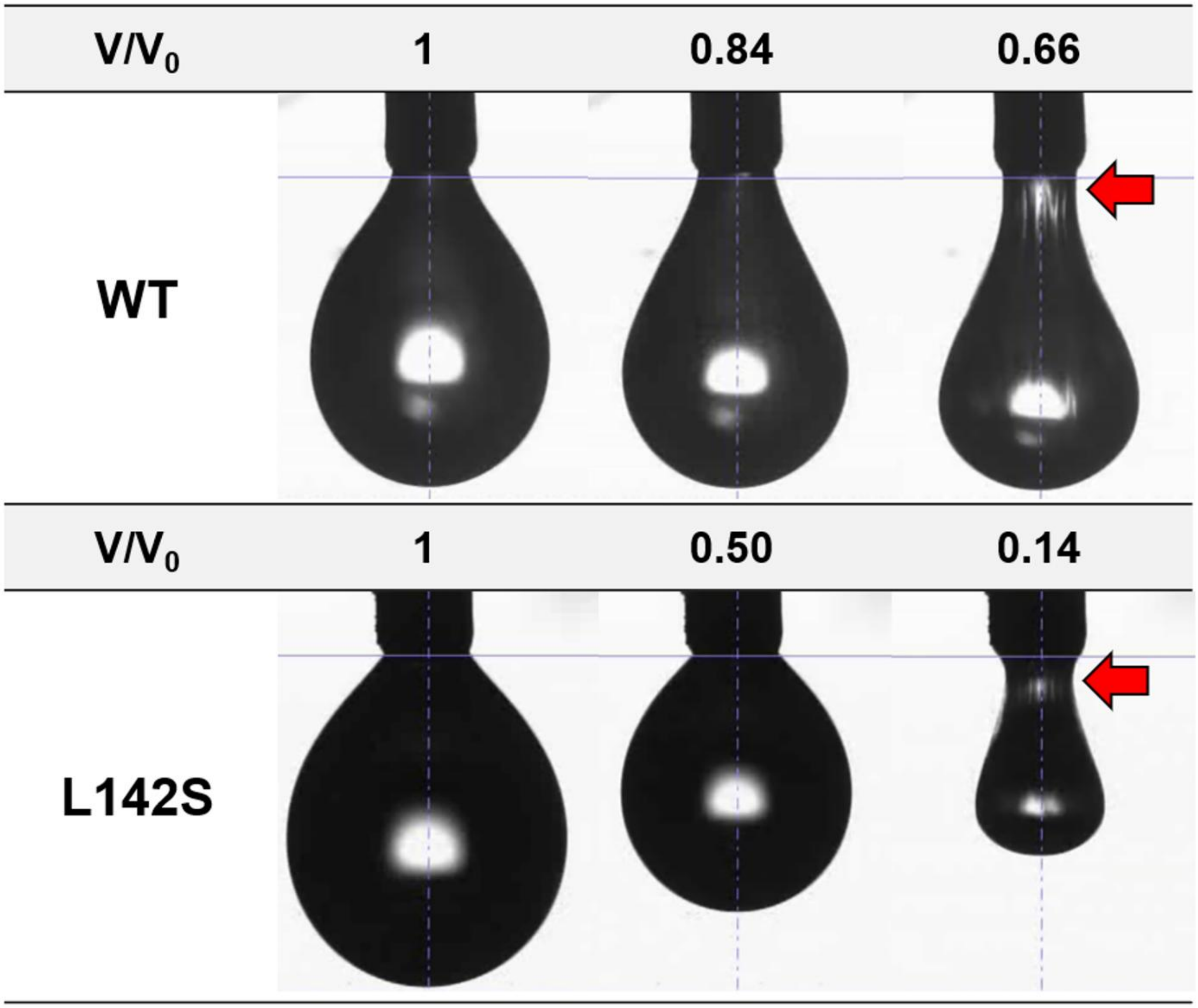

Compression ratios of RolA-WT and RolA-L142S droplets suspended in air. The concentration of RolA was 100 μg/mL. Clear buckling (arrows) was observed in RolA-L142S droplets at compression ratios (V/V_0) smaller than those of RolA-WT.

Figure S6

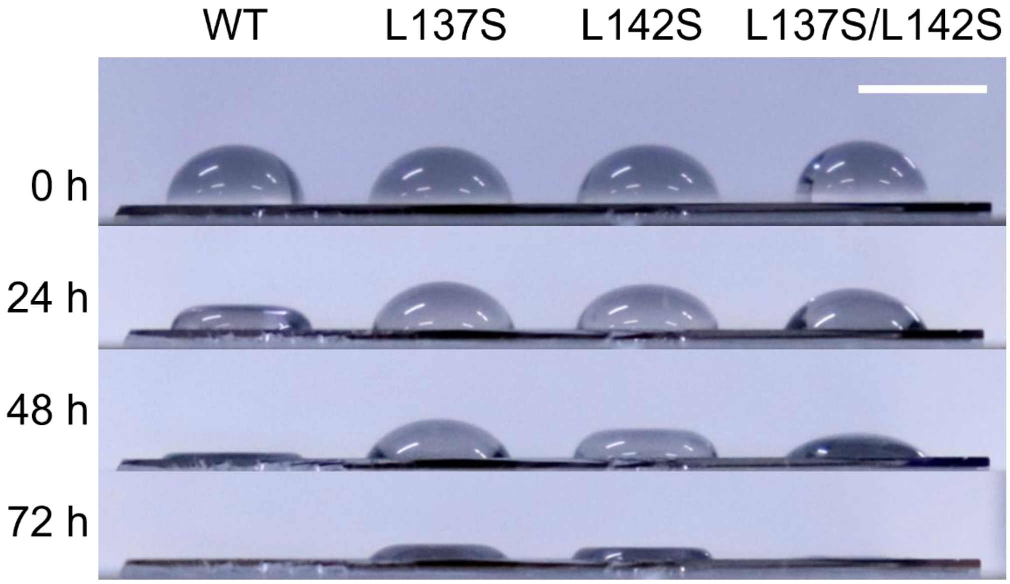

Flat droplets of the solutions of RolA-WT and its mutants. Plateaus were observed at the tops of all droplets. Scale bars = 2 mm.

Figure S7

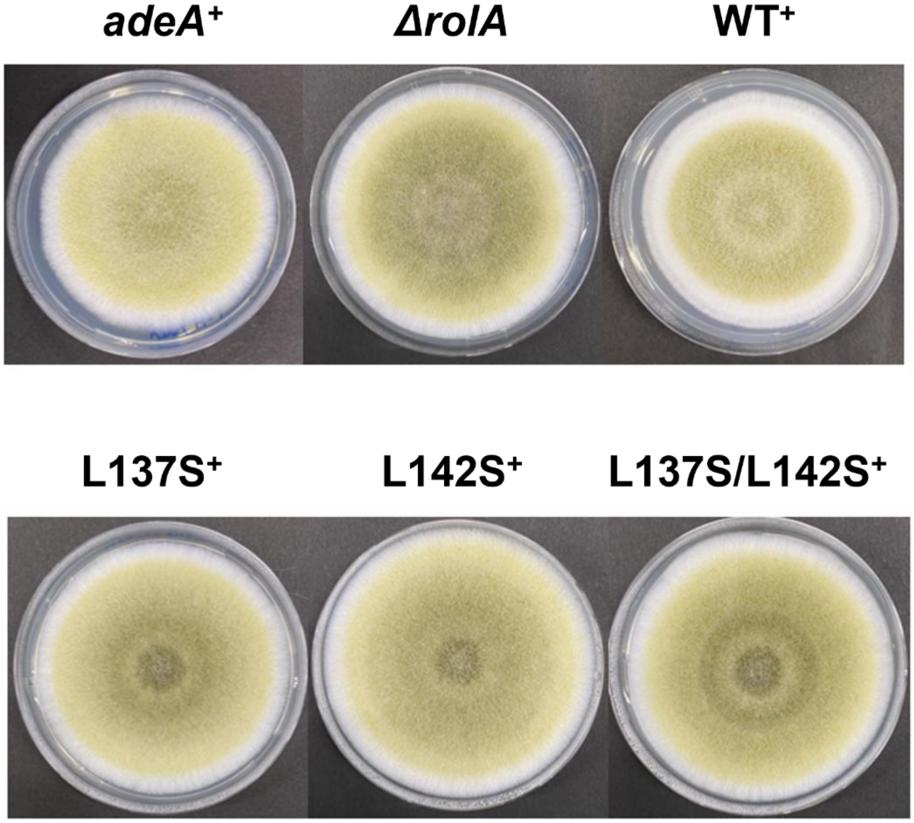

Colony morphology of *A. oryzae* control (*adeA*$^+$), deletion (*ΔrolA*), and complemented strains on potato dextrose agar.

Table S1 Strains used in this study.

Strain	Parent strain	Genotype
RIB40		Wild-type strain (=ATCC42149)
eno-hyp WT	NSID-tApEnBdIVdV2	*niaD*$^-$, *sC*$^-$, *adeA*$^-$, Δ*argB*::*adeA*$^-$, Δ*ligD*::*argB*, Δ*pyrG*::*adeA*, Δ*tppA*, Δ*pepE*, Δ*nptB*, Δ*dppIV*, Δ*dppV*::*pyrG*, *pNEN142-AorolA*::*niaD*
eno-hyp L137S	niaD300 (*niaD*$^-$)	*niaD*$^-$, *pNG-eno-hyp L137S*::*niaD*
eno-hyp L142S	niaD300 (*niaD*$^-$)	*niaD*$^-$, *pNG-eno-hyp L142S*::*niaD*
eno-hyp L137S/L142S	niaD300 (*niaD*$^-$)	*niaD*$^-$, *pNG-eno-hyp L137S/L142S*::*niaD*
adeA$^-$		*niaD*$^-$, *sC*$^-$, Δ*ligD*::*sC*, Δ*adeA*::*ptrA*
adeA$^+$	*adeA*$^-$	*niaD*$^-$, *sC*$^-$, Δ*ligD*::*sC*, Δ*adeA*::*ptrA, adeA*$^+$
Δ*rolA*	*adeA*$^-$	*niaD*$^-$, *sC*$^-$, Δ*ligD*::*sC*, Δ*adeA*::*ptrA*, Δ*rolA*::*adeA*
RolA WT$^+$	Δ*rolA*	*niaD*$^-$, *sC*-, Δ*ligD*::*sC*, Δ*adeA*::*ptrA*, Δ*rolA*::*adeA*, *rolA*$^+$, *RIB40p-AorolA WT*::*niaD*
RolA L137S$^+$	Δ*rolA*	*niaD*$^-$, *sC*$^-$, Δ*ligD*::*sC*, Δ*adeA*::*ptrA*, Δ*rolA*::*adeA*, *rolA*$^+$, *RIB40p-AorolA L137S*::*niaD*
RolA L142S$^+$	Δ*rolA*	*niaD*$^-$, *sC*$^-$, Δ*ligD*::*sC*, Δ*adeA*::*ptrA,* Δ*rolA*::*adeA*, *rolA*$^+$, *RIB40p-AorolA L142S*::*niaD*
RolA L137S/L142S$^+$	Δ*rolA*	*niaD*$^-$, *sC*$^-$, Δ*ligD*::*sC*, Δ*adeA*::*ptrA*, Δ*rolA*::*adeA*, *rolA*$^+$, *RIB40p-AorolA L137S/L142S*::*niaD*

Table S2 Primers used in this study.

Primer	Sequence (5′ to 3′)
L137S Fw	GGCGACTCCGTCGGTATTGGTCTTCC
L137S Rv	CCGACGGAGTCGCCATCCTGTGATTG
L142S Fw	ATTGGTTCCCCTTGCATCGCTCTCGGC
L142S Rv	GCAAGGGGAACCAATACCGACGAGGTC
L137S/L142S Fw	GGCGACTCCGTCGGTATTGGTTCCCC
L137S/L142S Rv	ACCGACGGAGTCGCCATCCTGTGATTG
pNEN142-enoA-RolA Fw1	TCTTCGCTATTACGCCAGCTG
pNEN142-enoA-RolA Fw2	CCCATAGGTGAGTTTGGTTG
pNEN142-enoA-RolA Fw3	GTAGGGATATCATCGCATGGC
pNEN142-enoA-RolA Fw4	CCTCCCTTTCCGTCTCTTTTC
pNEN142-enoA-RolA Rv1	GGACGAGGTCCAACACTCAC
pNEN142-enoA-RolA Rv2	ATTTGGTCGATGAAGACGACTC
pNEN142-enoA-RolA Rv3	GCAACTCGCTTACCGATTACG
pNEN142-enoA-RolA Rv4	CCCCGTAGAATCACGAATGAG
In-Fusion Fw2	ATCTTCCGGTATGGCTGTTG
In-Fusion Fw3	GAAGCAGTGAGATGGATGAGG
In-Fusion Fw4	CCAGGGTCTTGGTCTCTTGG
In-Fusion Rv1	GGACGAGGTCCAACACTCAC
In-Fusion Rv2	ATTTGGTCGATGAAGACGACTC
In-Fusion genome Fw	AAACGACGGCCAGTGGGACCGTGCAGTAGTAGAGTTC
In-Fusion genome Rv	ATGCCATATGACTAGGTGATGGTGGCTTTGGTTTTG
In-Fusion InversePCR Fw	CTAGTCATATGGCATGCAGTTC
In-Fusion InversePCR Rv	CACTGGCCGTCGTTTTACAAC
AorolA-top-f	GGACCGTGCAGTAGTAGAGTTCCTAAC
AorolA-top-r	CGCGTGCGCAGGAGTGTTTGTTGTGTGATGGTGGCTTTGGTTTTGAACGAG
AnAdeA-f	CAACAAACACTCCTGCGCACGCG
AnAdeA-r	GGTACCTGATGGCCTCGCAGATAAA
AorolA-bottom-f	GTTTATCTGCGAGGCCATCAGGTACCGCGATTGCATTCGCGAAAATGGTAGCTC
AorolA-bottom-r	GTTCGGGGTTTTTTCTTTGGTGTGTACTTGGTGC
AoRolA-delta-L-chk-f2	TACCAAACGCGGGCACCAG
AoRolA-delta-R-chk-r2	AATCGGCGGATGAGTCG
AnAdeA-chk-f2	GGTAAACGAGCCGAGAGAATC
AnAdeA-chk-r2	GGTAGCAGAATTGTCTCCCATC
AoRolA-chk-f	ACCACCAAGCAGGCCG
AoRolA-chk-r	GAGCGATGCAAGGAAGAC
Ao Histone-RT Fw	ACGTCGTCTATGCCCTCAA
Ao Histone-RT Rv	GGAAGGACAACCGACGCG
Ao kexB-RT Fw	GCGACATCAGTGTGGAGTTG
Ao kexB-RT Rv	GACCGTCCACTTTCCAACAC
Ao RolA-RT Fw	TGCACCAACATCCCTGTTATCC
Ao RolA-RT Rv	GGACTTGGTGTTCTGGCAGC

Table S3 Height, width, and length of rodlets and rod-like structures formed by hydrophobin RolA. All numerical values are in nanometers.

Substrate	RolA	π = 20 mN/m			π = 30 mN/m		
		Height	Width	Length	Height	Width	Length
Hydrophilic silicon	WT	2.63 ± 0.15	12.0 ± 1.83	76.0 ± 34.2	2.10 ± 0.40	12.0 ± 1.83	95.9 ± 35.0
	L137S	2.70 ± 0.33	12.4 ± 3.05	73.7 ± 38.5		–	
	L142S		No rodlets		2.78 ± 0.32	11.6 ± 2.10	93.0 ± 41.1
	L137S/L142S		No rodlets			–	
Hydrophobic silicon	WT	6.85 ± 0.36	19.9 ± 3.58	69.6 ± 26.3	7.96 ± 0.40	20.5 ± 2.11	89.4 ± 25.7
	L137S		No rodlets			–	
	L142S		No rodlets		6.87 ± 0.83	16.0 ± 2.54	96.4 ± 48.0
	L137S/L142S		No rodlets			–	
